# The evolution of technical prerequisites and local boundary conditions for optimization of mitral valve interventions—Emphasis on skills development and institutional risk performance

**DOI:** 10.3389/fcvm.2023.1101337

**Published:** 2023-07-21

**Authors:** Riccardo Cocchieri, Bertus van de Wetering, Jan Baan, Antoine Driessen, Robert Riezebos, Sjoerd van Tuijl, Bas de Mol

**Affiliations:** ^1^ OLVG Hospital, Amsterdam, Netherlands; ^2^ LifeTec Group BV, Eindhoven, Netherlands; ^3^ Amsterdam University Center, Technical University Eindhoven, Amsterdam, Netherlands

**Keywords:** mitral, minimally, invasive, percutaneous, risk, reduction, TMVR, MIMVS

## Abstract

This viewpoint report describes how the evolution of transcatheter mitral valve intervention (TMVI) is influenced by lessons learned from three evolutionary tracks: (1) the development of treatment from mitral valve surgery (MVS) to transcutaneous procedures; (2) the evolution of biomedical engineering for research and development resulting in predictable and safe clinical use; (3) the adaptation to local conditions, impact of transcatheter aortic valve replacement (TAVR) experience and creation of infrastructure for skills development and risk management. Thanks to developments in computer science and biostatistics, an increasing number of reports regarding clinical safety and effectiveness is generated. A full toolbox of techniques, devices and support technology is now available, especially in surgery. There is no doubt that the injury associated with a minimally invasive access reduces perioperative risks, but it may affect the effectiveness of the treatment due to incomplete correction. Based on literature, solutions and performance standards are formulated with an emphasis in technology and positive outcome. Despite references to Heart Team decision making, boundary conditions such as hospital infrastructure, caseload, skills training and perioperative risk management remain underexposed. The role of Biomedical Engineering is exclusively defined by the Research and Development (R&D) cycle including the impact of human factor engineering (HFE). Feasibility studies generate estimations of strengths and safety limitations. Usability testing reveals user friendliness and safety margins of clinical use. Apart from a certification requirement, this information should have an impact on the definition of necessary skills levels and consequent required training. Physicians Preference Testing (PPT) and use of a biosimulator are recommended. The example of the interaction between two Amsterdam heart centers describes the evolution of a professional ecosystem that can facilitate innovation. Adaptation to local conditions in terms of infrastructure, referrals and reimbursement, appears essential for the evolution of a complete mitral valve disease management program. Efficacy of institutional risk management performance (IRMP) and sufficient team skills should be embedded in an appropriate infrastructure that enables scale and offers complete and safe solutions for mitral valve disease. The longstanding evolution of mitral valve therapies is the result of working devices embedded in an ecosystem focused on developing skills and effective risk management actions.

## Introduction

This contribution describes the ongoing evolution in the field of the interventional treatment of the growing burden of mitral valve disease (1). Despite significant progress in the medical treatment of heart failure (2), timely interventional control of severe mitral regurgitation may significantly alleviate symptoms and have a sustainable positive effect on quality of life and life expectancy.

This evolution consists of three tracks:

1. The first evolutionary track constitutes the progress made in the field of interventional treatment of heart failure and mitral valve disease (e.g., the development of the state of the art in MVS and TMVI).
2. The second track is the evolution from idea to engineering and technical solutions, such as the implant itself, and the supporting technology such as imaging and auxiliary tools. Human factor engineering is a critical aspect influencing the device safety profile and the need for skills training. The next step is making the device available in an affordable way with a high degree of clinical success at acceptable risk.
3. The third track consists of creating and maintaining an ecosystem for innovations. This system is the habitat in which teams adopt surgical and percutaneous techniques. It is founded on an infrastructure of technical and professional support, skills development and institutional risk management, including shared decision making.

The first track will be described by means of a concise, but incomplete literature review which explores the development of a common treatment practice and clinical lessons shaped by the framework of clinical reports. The second track is a description of the R&D process and methods used in order to test innovative designs for structural integrity, reliability, predictable performance and usability, which are the major requirements for market certification. The third track covers the evolution within the Amsterdam cardiac ecosystem and describes the acquisition of institutional data.

## Introduction: Boundary conditions for innovation

Clinicians involved in technical innovations should be aware of some leading principles or strict boundary conditions; safety comes first and should even precede the promise that the claim of effectiveness is met:

1. There should never be any doubt that a percutaneous device carries an “a priori low” interventional risk compared to surgery, as it reduces injury and avoids intensive postoperative care. This should result in less discomfort, pain and anxiety for the patient.
2. This short-term risk reduction provides room for taking and accepting the potential risk of reduced effectiveness. Trading interventional risks and clinical outcomes is the core of medical and shared decision making by Heart Teams and patients.
3. It is a regulatory requirement that technical and clinical claims should be supported by pre-clinical testing and clinical studies, which should be shared with stakeholders in an accessible manner.
4. Operators should be aware of the strength and weakness of the technology they use, in terms of requirements of infrastructure, peri-operative care and skills levels of medical and paramedical personnel involved in the procedure.

Acceptation and incorporation of innovations depends on many factors, including affordability, marketing, effectiveness and importantly, usability. Feasibility testing verifies whether a technology or a device behaves as designed and achieves the intended effect; it describes the barriers and challenges under laboratory conditions and within controlled clinical studies with selected patients. After passing these tests, physicians and patients may trust the technical specifications and the structural integrity of the system: no software flaws and no loose parts or fractures should be expected.

Usability testing assesses the efforts and capabilities that are necessary before operators and their team can safely apply a new technology; secondly, it verifies the extent to which a relevant therapeutic goal can be achieved at acceptable risk. Usability entails the human factor interfaces (HFI) between a technology, the operator, and the patient. These features shape technical and clinical claims associated with the device or technology, and its indication for use.

Exposing a patient to surgery implies that the surgeon provides relief of mitral regurgitation (MR) at an acceptable perioperative risk, depending on timing of surgery and comorbidity. An experienced surgeon disposes of many techniques, including a bail-out with valve replacement and can adapt or integrate them, in order to achieve the desired result. In contrast to this, the operator performing a transcatheter mitral valve intervention (TMVI) can do no more than execute the prescribed actions for which he has been trained in order to achieve the highest chance of technical success. During the same procedure the surgeon has many options, which allow for a margin of optimization and adjustment. In other words, the level and intensity of control in surgery and transcatheter mitral interventions (TMI) are different: technology comes first in a transcutaneous procedure and skills first in surgery. Both technologies require a high degree of familiarity and experience with the preparatory and executive actions. In order to achieve a high chance of technical and sustainable therapeutic success, periprocedural imaging has become mandatory in both approaches (3–5). It all starts with optimal access to the mitral valve; a good exposure is essential for conducting appropriate mitral valve surgery (6) while bendability and access are critical for the transcutaneous device.

## Track one: Common clinical practice reporting

### Mitral valve surgery

The increasing volume of knowledge around the surgical treatment of mitral valve regurgitation is illustrated by the frequency of reports on this subject in scientific journals. Recent reviews, in particular the one by Nappi et al. (7), which is based on a thorough study of the literature and a meta-analysis of treatment options for mitral regurgitation, describe the way to deal with degenerative and ischemic mitral valve disease (3, 8, 9). With the certainty that mitral repair should be preferred in degenerative mitral regurgitation, despite increased early mortality, even mitral valve replacement is an acceptable solution if multi-level reconstruction of leaflets, chordae, annulus and papillary muscle cannot be achieved, especially where the chordal apparatus is preserved.

Although perioperative risks can be reduced to a low level, patient selection is essential in order to predict the elimination or significant reduction of MR. Equally important is the “a priori” likelihood of the degree to which the effect of the sequelae of longstanding MR can be halted or reversed. The presence of pulmonary hypertension, impaired left and right ventricular function and tricuspid valve regurgitation largely determines clinical benefits in terms of quality of life and life expectancy; these also apply in TMVI and will be discussed later (7).

The evolution of mitral valve surgery has yielded decades of experience of surgical technique, imaging support and short- and long-term outcomes(10); the timing and selection of surgery has been the subject of debate and studies (11, 12). The surgical experience has produced predictable outcomes and enabled shared decision making. Thorough informed consent regarding benefits and risk, based on a multidisciplinary heart team decision, the concept of heart team decision making has become the standard of care (13, 14).

### Transcutaneous mitral valve interventions

The development of TMVI is driven by the avoidance of open-heart surgery, postoperative care and with the aim of decreasing patient discomfort. It is estimated that impaired LV function, older age and comorbidity prohibit surgery in half of the patients with primary severe MR (11). Edge-to-edge clip devices are most used for transcatheter edge-to-edge repair (TEER), and recently the Abbotts Tendyne TMVR device (Abbott Vascular, Santa Clara, CA) has been approved for clinical use in Europe. In addition, TEER may also be considered in patients with functional MR (15). However, despite its initial low-risk implantation and encouraging results (16), the clinical results of TEER are affected by the same limitations as surgery: residual MR, presence of TR, presence of atrial fibrillation (AF), impaired LV and RV function, pulmonary hypertension, fibrosis of the myocardium, and patient-related comorbidity (17–25). Large trials have resulted in conflicting evidence concerning the benefits and sustainability of clinical effects, but the general conclusion has been that careful patient selection is essential in order to offer patients a predictable clinical benefit (26–30).

The number of patients receiving TEER is increasing, probably due to better heart failure work-ups, the predictability of TEER success and failure, and increasing experience (31). In addition, other types of device to reduce the annular circumference have become available, although the reported clinical experience remains limited (32–35).

While based on a completely different concept, the increasing experience with TMVR is showing promising results and despite challenges regarding sizing, durability and appropriate indication, it has solved the issue of the recurrence of mitral regurgitation. Although repair seems to be the preferred solution for MR in MVS, TMVR is advocated as a concise and complete solution even in secondary MR (36, 37) as it may be a substitute for MVR with preservation of the chorda (30, 38, 39).

Summarizing, the evolution of mitral valve interventions has been marked by many well conducted studies, which indeed have provided many lessons learned and yet to be learned at the crossroads of MVS and TMVI (40, 41). Minimally invasive mitral valve surgery (MIMVS) and Transcatheter mitral valve interventions (TMVI) are adjacent technologies which can be applied at a low perioperative risk in selected patients. Both approaches rely on state of the art imaging technology and computational support (4, 42–47), which is quickly assuming an important role, even in conventional surgery (48–50). Additional findings in preoperative patient characteristics such as exercise capacity, body mass index, peripheral vascular disease and lung capacity, may affect short- and long- term outcomes for both technologies (51–55) and should be probably taken into consideration when discussing the indication for a certain treatment.

## Defining clinical boundary conditions

A growing number of publications have become available thanks to computer sciences and bio-statistics, which enabled registries, long-term single institutional experiences, meta-analysis reviews. In retrospect we can agree that starting with Valve academic research consortium definitions for TAVI and mitral therapies (VARC and MVARC) (56, 57) and the simultaneous creation of national and international registries, the standardization of definitions of success, failure, clinical events and relevant parameters, has become a matter of fact. This is an evolutionary achievement which should not be underestimated. Initiatives for clinical research and reporting have increasingly benefitted from the growing force of computer science and sophisticated biostatistics. This allows the creation of very large data sets based on clinical experience with thousands of patients, but also in-depth analysis of smaller groups of patients with the possibility of combining biomarkers, imaging and hemodynamic data. It also enables rather reliable use of propensity matching and creation of useful predictive modelling. In other words, developments in surgical technique and TMVI devices come first, but assessing their usability, safety and clinical effectiveness in controlled and undisputed way by using computer power is the indispensable driver for rapid adoption in many centers.

The message coming from clinical practice reports the is that technology helps despite some disclaimers regarding careful patient selection, a steep learning curve, and the fact that more research is needed. Nevertheless, as a matter of fact, publication of clinical data provides building blocks for market approval of technology, professional standards and guidelines; there is no doubt that leadership obliges physicians to publish.

One may question the impact of the many publications on mitral valve disease on common practice within intervention centers for mitral valve disease.

The dissemination of experience by means of publications carries a substantial degree of “empiricism” and “lessons-to-be-learned”. However, when the reference frame is clear, the performance data provide a benchmark for individual teams and institutions. The relevance may depend on study methodology (multicenter or single-center experience), or focus on a particular subclass of treatment, or on robustness created by the support gained from other publications.

Availability of computer power, hospital information systems and support staff, enables internal quality monitoring and benchmarking. From this perspective publications are the tip of the iceberg. This availability enables cardiac teams to publicly share performance data by means of registries or by inter-institutional connections.

In absence of RCTs comparing techniques and procedures, one may argue that computer power provides a false substitute for the failure of organizing powerful comparative studies. However, one may also argue that RCTs should be saved to solve relevant debates regarding treatment outcomes based on disruptive technology with a large clinical and cost-effectiveness impact; especially, in case strong circumstantial evidence from sources such as registries is available. Technical improvements develop faster than RCTs can be organized, while the proportionality between costs and clinical relevance is missing.

Over time, the multidisciplinary Heart Team has become an imperative boundary condition as it makes decisions based on internal and external performance. This requires a solid and accessible institutional database structure within the hospital information system. Input by a data analyst is indispensable in order to generate “experience-based” and patient-tailored advice. In addition, skills development and a system of perioperative risk management may reduce adverse event rates (41, 55–60).

Institutional risk management starts with Heart Teams whose function should be to continually compare the outcomes of medical treatment, surgical and catheter interventions. Computer science and the availability of an hospital data infrastructure greatly determines the impact of Heart Teams and appropriate patient selection.

## Track two: Biomedical engineering from invention to application

Biomedical engineering covers many specialties such as computer-assisted design, materials, surface interaction, strengths analysis, biocompatibility, computational description of functionality and human factors engineering (61).

The design of a technology is the most dominant factor in determining the total product life cycle (TPLC) of a medical device. The concept of TPLC includes feedback, sustainability and the need for iterations. The relevance of TPLC to the design and risk management of medical devices is addressed excellently in two review papers by the group of Vassiliadis (62, 63). The development from idea to design, and from there to a usable product, depends heavily on choices made at the early design phase. Questions to be addressed are which problem to solve, interior and exterior shape, composition of materials and dealing with aspects related to the final use: user-friendliness, prevention of technical failure and prevention of human error. Human factors engineering (HFE) is an engineering discipline which focuses on ergonomics and processes that ensure ease and safety of use. According to Carayon, the understanding and anticipation of human error provides the basis for safe and reliable use of medical technology (64, 65). This understanding starts with identifying performance obstacles and improving system resilience by preparing for the unexpected and for failure. These obstacles are usually addressed in bail-out procedures and can be trained with simulators. As a part of design and development, HFE should focus on the usability of technology, human error prevention, clinical performance, and resilience. This implies that during the design phase of a new device, characteristics of the operating room/catheterization laboratory, such as dependence on supporting technology and hospital infrastructure should be taken into account from the very beginning (66, 67).

However, the expanding technical requirements for MIMVS and MVI, in particular imaging, robotics and the many software and hardware interfaces, put high demands on operators in terms of skills development, spatial awareness and readiness to respond inside and outside the boundaries of the given task (41, 62, 68–70).

Paradoxically, technology improves and creates chances for a better treatment, but at the same time puts higher demands of the operators. While one of the characteristics of a new percutaneous device should be the reproducibility of its results, even in less experienced hands, in the early phase of the development is important to look for the best qualified operator with the highest skills. At the same time, at the beginning of the TPLC an experienced R&D team must stand up to shape the innovation.

### Proof of safety and reliability

An additional complicating factor is the heavy burden of regulatory proof required to provide technical and clinical evidence that the specific technology is safe and effective, especially in the long-term and in the case of high-risk implants for mitral valve disease (71). One way to control the very costly need for trials is to make certain that the research and development department (R&D) should obtain the user's input at the very early phase in order to guarantee a very low technical and procedural failure rate (72, 73). Acting in this way can prevent costly design adjustments, which in retrospect were due to evident shortcomings of the technology.

The R&D track is time-consuming and even before launching the final product on the market, proving the product's compliance with the safety and efficacy standards needed for market approval is expensive (62). Development from invention to a certified product ready for marketing has many procedural steps: the incremental costs connected to each subsequent step reflect the growth in value of technology and the potential success that your product will have before launched. The credibility that your company will offer to your future customers is also based on the quality of those tests.

**Figure 1:**
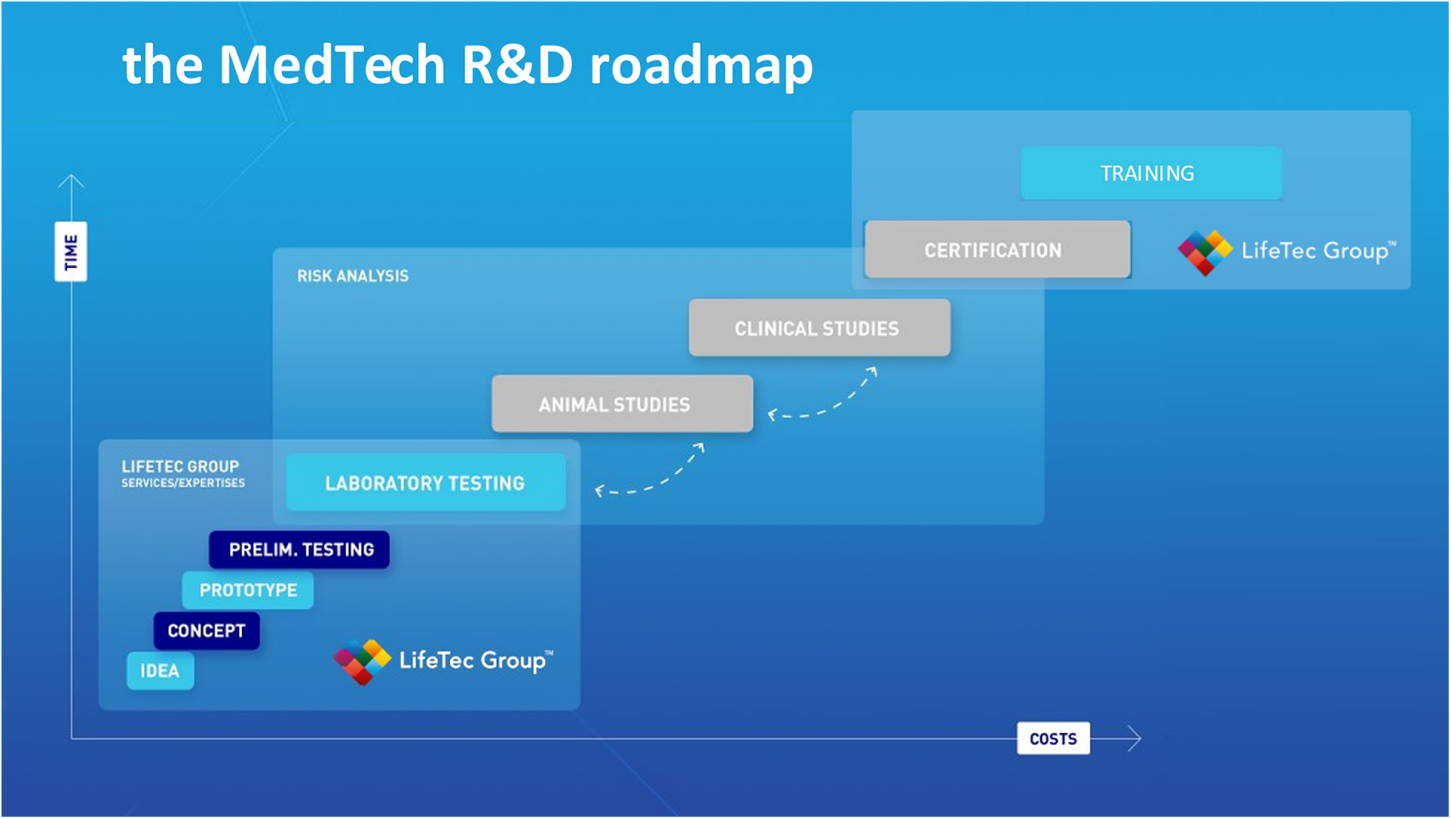
This illustration is used by lifeTec group to explain and emphasize the importance of solid and comprehensive preclinical studies including computational modeling, virtual reality, use of in-silico set-ups and finally the use of a biosimulator with active or passive working hearts.

Figure 1 describes the critical steps in R&D development; it shows that costs incurred in the preclinical phase prior to animal testing are the lowest. This explains why it is preferable to gain as much knowledge as possible in terms of risks of failure, usability and human error as well as technical reliability in the laboratory testing phase prior to animal testing. The use of bio-simulators with working animal or human hearts means a step down in the costs that are usually made at the animal and clinical study phase. Test combined with the entire imaging modality range and adaptive manipulation may be even more complete and effective and guarantee more reproducible results.

Despite the promises, there continues to be a substantial risk of technology failure or uncertain marketing, and this risk even increases at each step toward the final goal of first human implant and market approval. Therefore, solid preclinical testing of a freeze design is essential and this should be the first working prototype put into the hands of future users. Before testing the freeze design in an in-silico or ex-vivo setting, issues about patents and intellectual property rights should have been settled. The laboratory setting should confirm that the product does what it is supposed to do. Based on these findings, animal welfare committees can then be convinced that animal tests are justified in order to confirm the intended clinical use. At this stage of the R&D process, major technical obstacles for imaging, delivery system and implant should also have been addressed.

**Figure 2:**
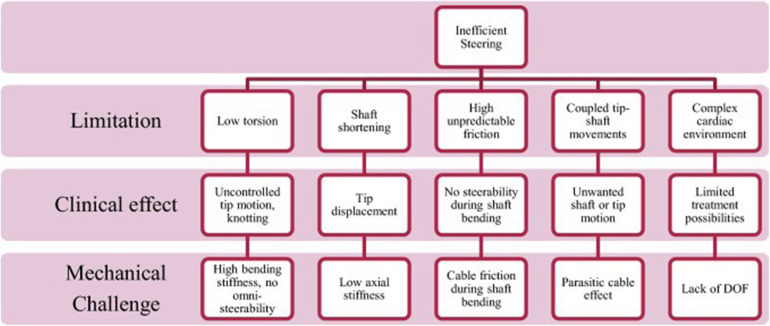
Shows parameters relevant for usability testing by clinical experts.

**Figure 3:**
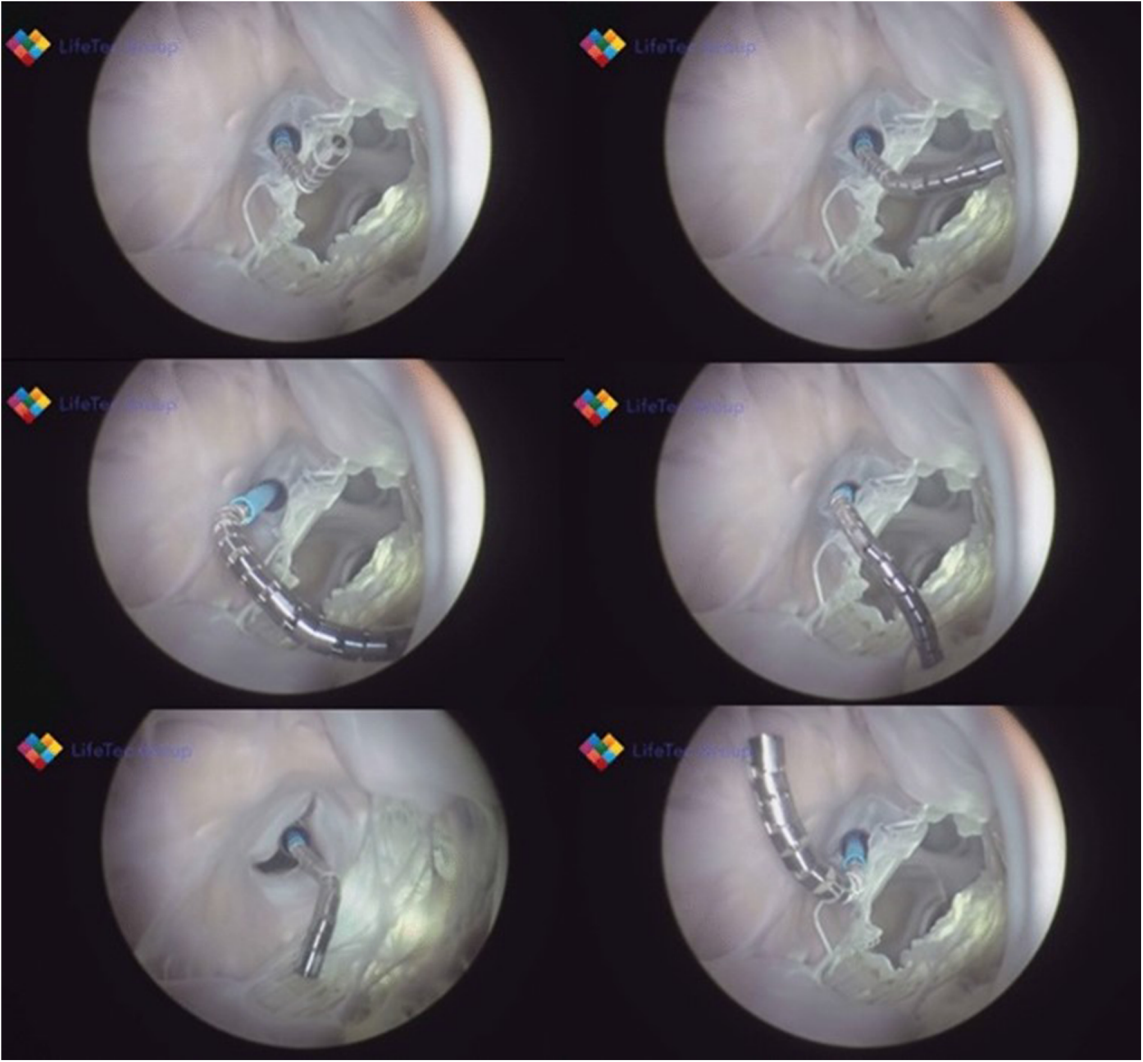
Shows various frames from a passively beating pig heart. Images are acquired endoscopically and they visualize the atrial septal perforation and bendability of the catheter in relation to the mitral valve.

**Figure 4:**
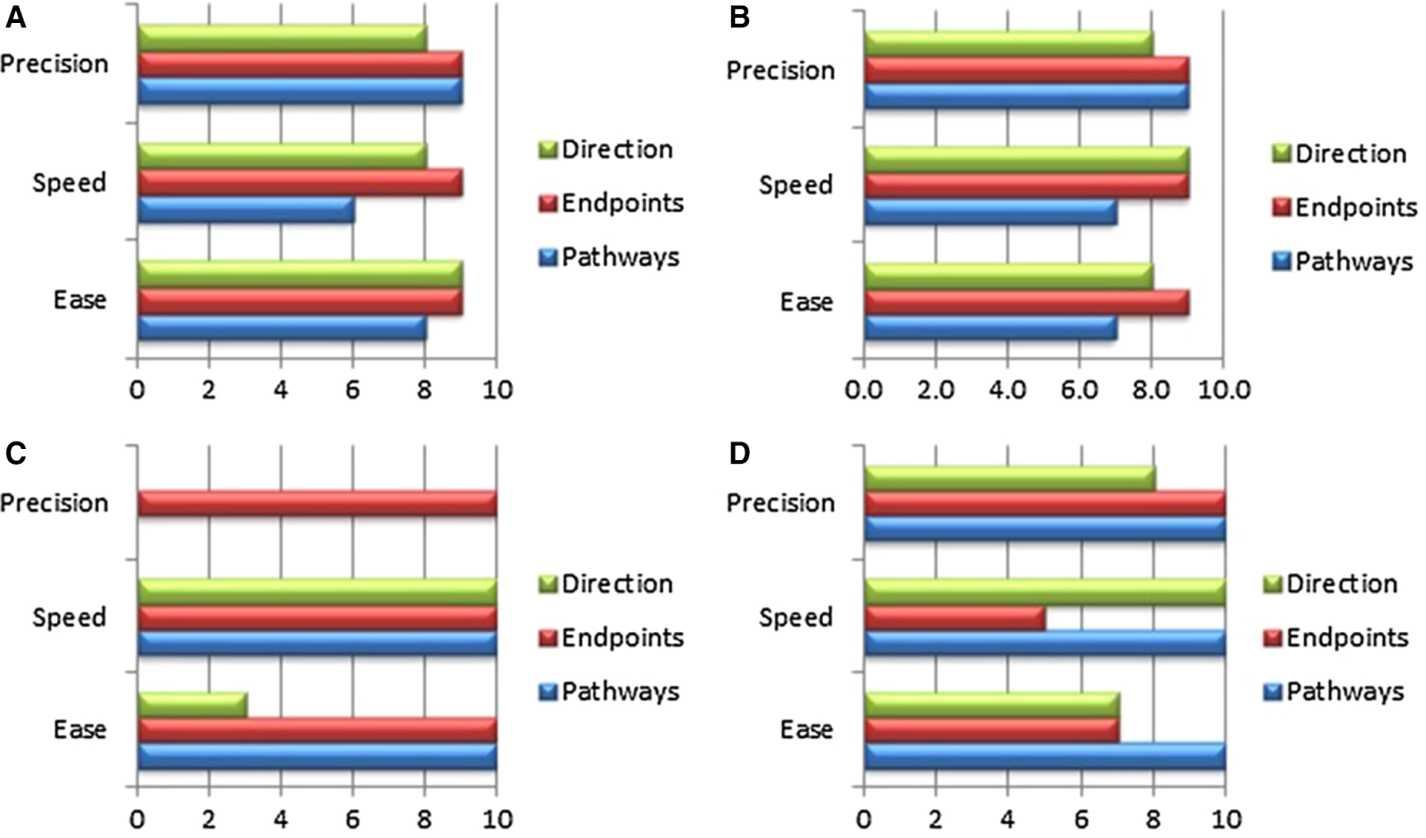
The panels (**A**–**C**) show essential components, which were tested in passive beating pig hearts and a living working slaughterhouse heart. Ali A, et.al. First Expert Evaluation of a New Steerable Catheter in an Isolated Beating Heart. Cardiovasc Eng Technol. 2020 Dec;11(6):769–782. doi: 10.1007/s13239-020-00499-3.

In the case of mitral valve therapy, a comparison with an existing or standard device therapy may be conducted. Various models are available to simulate the expected final result; steerability and bendability of catheters are essential requirements for TMVI devices. Ali et.al. have developed an expert preference test (Physicians Preference Test—PPT) for catheters in a cardiac biosimulator, with a passively beating heart (73). Figures 2–4 show those parameters that can be measured, including ease and frustration.

In such a Physicians Preference Test (PPT)*,* which usually comprises a focused inquiry for operators on the actual use of a given prototype, various aspects of the procedure, the handling of the device and the operator's experience with the innovation, are compared with the standard reference procedure (63). It is valuable to have the intended benefits related to the use of a given device confirmed, but even more important that specific expected and unexpected risks are revealed. The feedback coming from the PPT may result in a design change, which is costly, but always cheaper that dealing with failure at a later stage.

### The role of a cardiac biosimulator (CBS)

The R&D team should critically evaluate the subsequent prototypes during one or more process steps in the R&D cycle, which are also the first steps of TPLC. Timely verification of design aspects covered by HFE minimizes development risks and the need for unplanned design iterations after initial use in patients. Some degree of operational and clinical uncertainty will always remain, despite an optimal design and thorough testing in accordance with the mandatory regulations. Despite removal of obstacles to performance and facilitating simple error-proof handling to assure safety and reliability, a certain amount of unpredictability will follow the journey toward the final product. Simulators can be helpful in the “pre-freeze” design and the prototyping phase by providing a complete and realistic testing environment (74–76).

**Figure 5:**
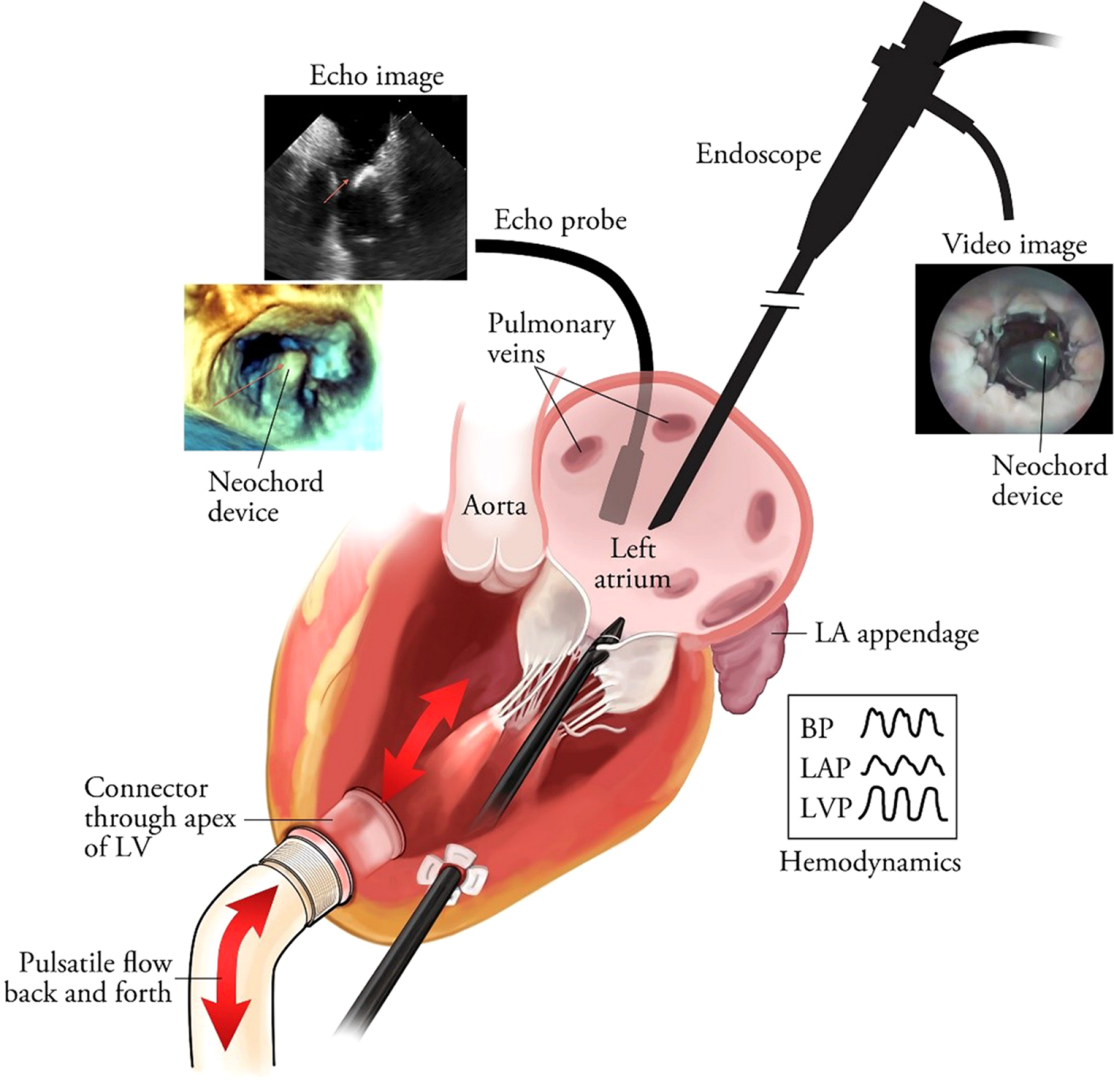
The schematic overview of the passively moving heart simulating working heart conditions in conjunction with aortic and mitral valve function. The standard set-up is MRI-compatible. In this illustration the frames show images obtained by echocardiography and videoscope in combination with hemodynamic data collection. The latter also enables high-speed recordings of valve function. Leopaldi et al. The dynamic cardiac biosimulator: A method for training physicians in beating-heart mitral valve repair procedures. J Thorac Cardiovasc Surg. 2018 Jan;155(1):147–155. doi: 10.1016/j.jtcvs.2017.09.011.

The availability of a cardiac biosimulator (CBS) with a passive moving heart addresses many aspects of technology testing, which is usually conducted by means of animal testing (Figure 5). Ali et.al. found that the passive beating heart provides the best platform (73). We describe in more detail the use of the LifeTecCBS (L-CBS), which has been designed to incorporate slaughterhouse animal hearts or specially treated human cadaver hearts. The use of this device may reduce the need for animal testing and help to better define research objectives for animal testing where living animals are required for regulatory approval.

The L-CBS can be used in a real-life operating room or catheterization laboratory environment, fully equipped with all modalities of echocardiography, biplane fluoroscopy, CT scan and MRI (76). The combined use of L-CBS and animal testing enables risk control and technical reliability scenarios and may be used together with other tests to predict a safe and effective execution of the procedure, relying in the meanwhile on live cardiac imaging (76).

### Challenges with device design for mitral valve disease

There is no “one-size-fits-all” solution for mitral valve disease. Unlike devices required for TAVI, transcutaneous devices for mitral valve repair are difficult to develop as implanting a device in a straight and stiff tube like the aorta tends to be easier than fixing a biological valve within a complex dynamic structure like the mitral apparatus. In TAVI, the technical result of the restoration of the valve function has an instantaneous beneficial effect. In TMVI, reduction of mitral insufficiency does not imply the automatic relief of symptoms. In mitral valve disease, several interdependent structures have to be addressed for optimal success: valves, annulus, chordae, papillary muscle and left ventricle. A percutaneous procedure usually addresses one or two structures and the cost of a single device is already high.

From this perspective, Transcatheter mitral valve replacement (TMVR) seems a promising and complete solution. It does not have the limitations of all the available mitral repair devices and mostly offers an outstanding degree of freedom from recurrence of MR and paravalvular leak (77). It spares the chordal and papillary muscle apparatus which in turn favors restoration of the LV function. Worldwide, numbers of implantations are unfortunately still low at probably around 2,000 cases and include the approved Tendyne and other non-CE/FDA-approved TMVR devices.

Currently, TMVR in native valve still suffers from the limitations of optimal sizing, interaction with adjacent structures [including coronary arteries and left ventricular outflow tract (LVOT)], and the side effects of an implanted artificial valve, mostly due to high risk of thromboembolic complications (78).

Research based on simulations with L-CBS and a human cadaver heart could potentially help in finding a better implantation technology that better adapts to the shape of mitral annulus. In summary, usability testing in the pre-clinical phase assesses design, safety and user-friendliness from the perspective of human factor engineering. It yields valuable information to select operators with an appropriate set of skills to be trained; it also defines boundary conditions for teams and hospital organization to ensure a safe use. The preclinical methods can be used for training, maintenance of skills and monitoring device performance.

## Track three: Institutional prerequisites for innovation

### The Amsterdam cardiac ecosystem

The two Amsterdam cardiac centers of OLVG Hospital and Amsterdam University Medical Center (AUMC) provide cardiac surgery and interventional cardiology services for a region with approximately 3.5 million inhabitants. Both centers offer a complete track for residency training in cardiology and cardiac surgery. Both are large general hospitals providing full-track training for nurses, paramedics and residents in many disciplines. The AUMC hosts a medical school and basic science departments in the same building, including departments of experimental cardiology, experimental radiology and biomedical engineering and medical physics.

**Figure 6:**
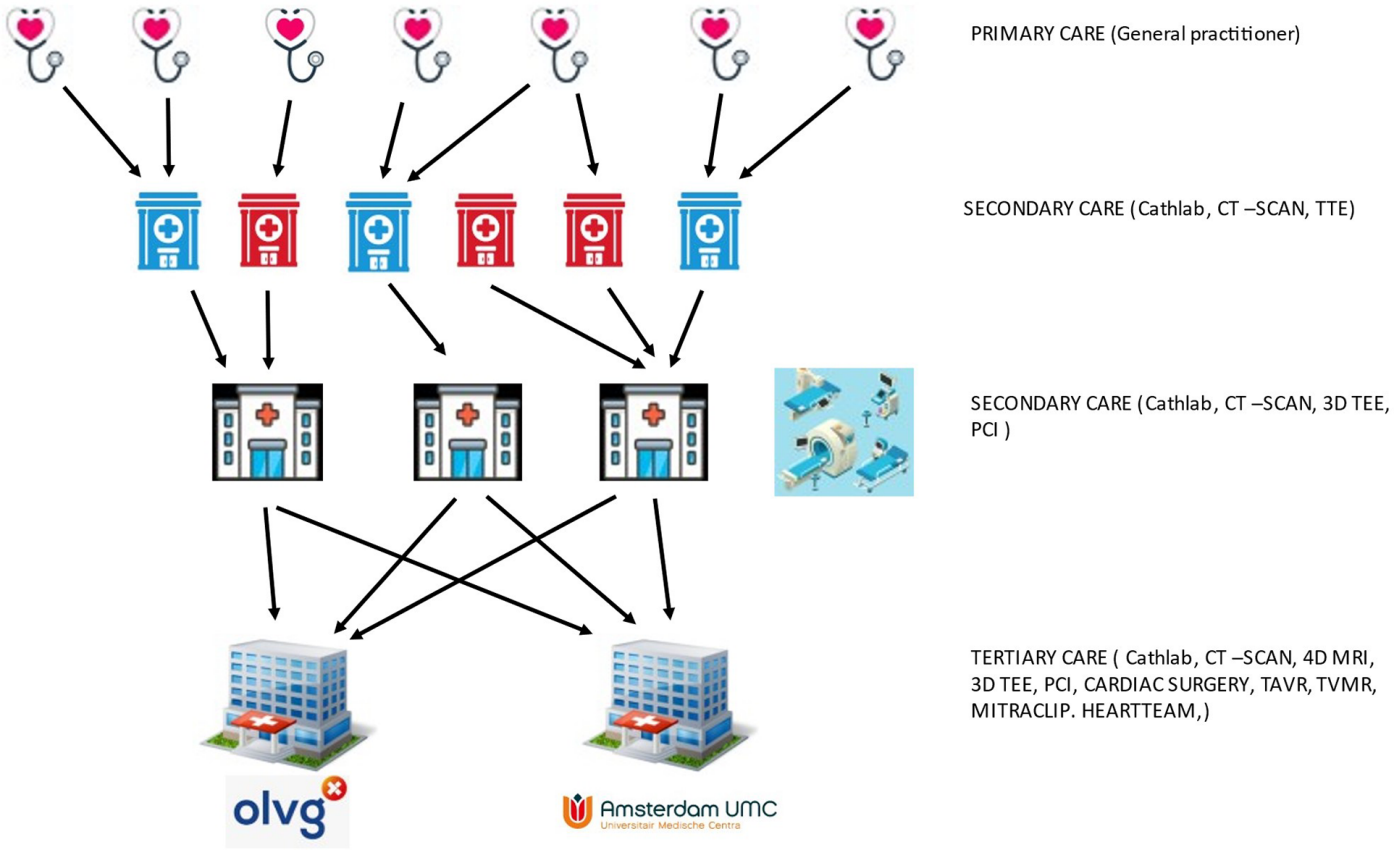
Schematic representation of referral paths and primary to tertiary cardiac care in Amsterdam.

The OLVG hospital is exclusively funded by health care insurers, while the AUMC also receives considerable contributions from the Netherlands Ministry of Education, Culture and Science (Figure 6).

Referrals for an expert opinion on mitral valve disease are usually made by second tier hospitals, but as teaching hospitals, the AUMC and OLVG do host large general cardiology practices as well. Over the years, this has developed into an organic growth model to fulfill the expanding demands to meet excellence and produce high-quality scientific output that results in numerous publications (79). However, even large cardiology and cardiac surgery departments must establish a way of making a sub-selection of specialties and patient case-mix. Both hospitals based their choices on the answers to the following questions:

1. What is new?
2. Does it fulfill an unmet, but significant medical need?
3. Is this already a focus of care and research?
4. Are the supporting infrastructure, expertise and skills available?
5. Can we make human and financial resources available over a longer period?
6. After a start-up period, will it fit into a reimbursement program?

Apart from the historical generic growth of services, this type of policy-making has resulted in a distribution of services between the two cardiac centers. The presence of infrastructure, qualified personnel, a quality assurance system and endorsement by health care insurers is mandatory.

In both hospitals TAVI required the upscaling of infrastructure including heart team commitment, and guidelines resulted in uniform practice and outcomes.

In combination with the presence of adequate infrastructure, this guarantees an optimal level of care. Health care insurers have explicitly stated that the numbers and quality of interventions should be sufficient to guarantee an adequate level of specialist care. In the meantime, the same insurers have required that patients should be able to choose between the AUMC and OLVG hospitals. This is not only to be able to offer different options to their customers, but especially to be able to negotiate pricing based on value-based health care principles (80) and in order to prevent monopolies in the health market.

### Adoption of Mitraclip

**Figure 7:**
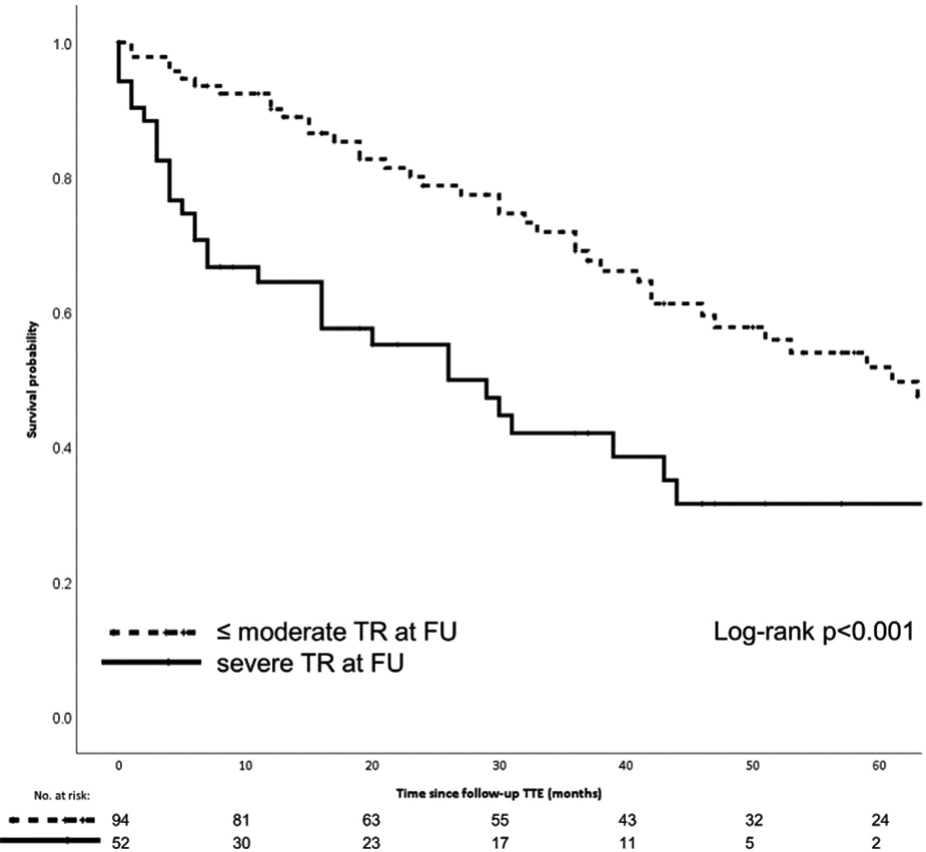
The Kaplan-Meier graphic shows the 5-year survival probability for patients who underwent Mitraclip repair with and without serious residual tricuspid valve regurgitation. Meijerink F, et al. Tricuspid regurgitation after transcatheter mitral valve repair: Clinical course and impact on outcome. Catheter Cardiovasc Interv. 2021 Sep;98(3):E427-E435. doi: 10.1002/ccd.29464.

The Mitraclip device for TEER received its CE mark in 2008 after the first implantation in 2003 (81). Since 2009, interventional cardiologists at AUMC have played an active role in the CE post-marketing study and have increased the number of implants to currently approximately 50 per year. Despite the technical improvements that different generations of Mitraclip have brought, general anesthesia is still required to perform the procedure. Evaluation of clinical outcomes has resulted in multi-center reports and single-institution reports on specific findings associated with TEER. In particular, the presence of severe TR was shown to affect the long-term survival after TEER, despite a low peri-interventional risk (Figure 7).

Despite the discordant messages that came from various RCT studies, Mitraclip technology has been implanted in more than 150,000 patients (Mitraclip website) and has found its way into the latest guidelines, with a IIB indication in secondary MR (15, 29, 40).

**Figure 8:**
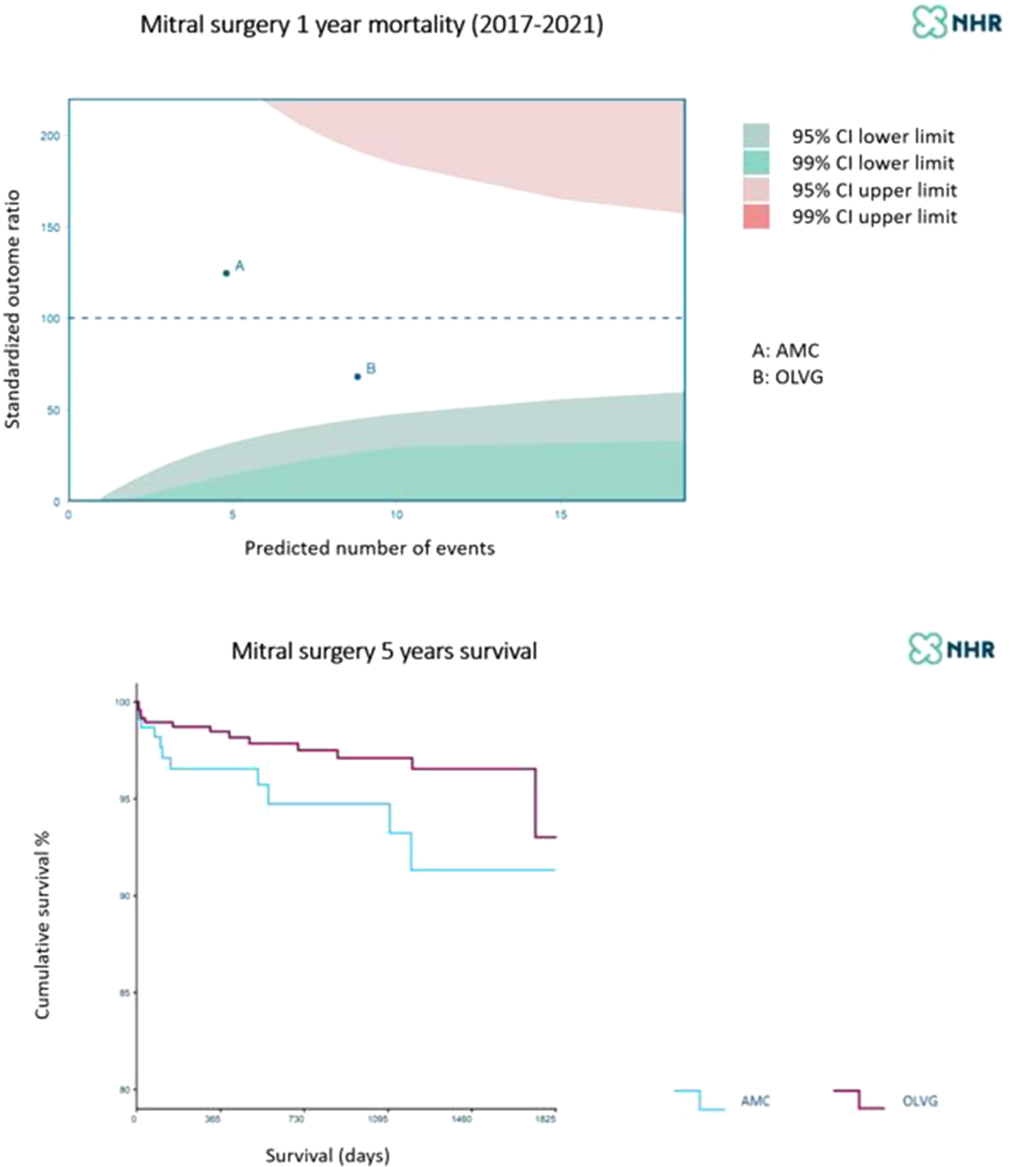
One-year and five-year mortality of mitral valve surgery at A = AUMC (blue) B = OLVG (purple). Patient selection and perioperative mortality make a difference while long-term survival is rather similar. Courtesy of the NHR 2017–2021.

In summary, the work-up and execution of TEER appeared even more laborious than that for TAVR, the practice of which was best addressed by the AUMC cardiac center; although both cardiac centers have been shown to have sufficient experience with MVR and MIMVS, the OLVG hospital has increased the amount of procedures performed in a minimally invasive fashion, creating the largest program in the country (61) (Figure 8).

Although the prevalence of mitral insufficiency is higher than that of any other valvular disease, the number of candidates eligible for TEER or TMVR in comparison to TAVI also appeared limited, which has consequences for the efficient use of available resources.

The experience with TEER in the AUMC also shows that a device fix of the mitral valve partially results in fixing mitral valve disease and heart failure. As shown in Figure 7, the presence of TR and AF resulted in worse long-term survival (19, 82). The challenge to optimize TEER results also yielded clinically-relevant research and lessons learned (52, 82, 83). The publications show that single-institution experience may provide useful insights to aid the establishment a recognized quality of clinical practice. This can be interpreted as proof of sufficient infrastructure and team willingness to innovate and become committed to TMVR (84, 85).

Participation in the NHR registry and the ambition to play a role in the innovation process for mitral valve therapies has also forced both cardiac centers to focus on perioperative patient management. The results demonstrate the quality of participant's practices in terms of patient selection, execution of the intervention, and perioperative risk control, including responsiveness measured by failure to rescue (59, 60). Clinicians should realize how dependent they are on biomedical engineering and computer science (61, 84). Artificial Intelligence and machine learning are indispensable to channel and analyze realtime large datasets relevant for decision-making process. It will be hard to bring these technologies into routine and clinical practice but early AUMC experience looks promising (86–88).

### Readiness for innovation

**Figure 9:**
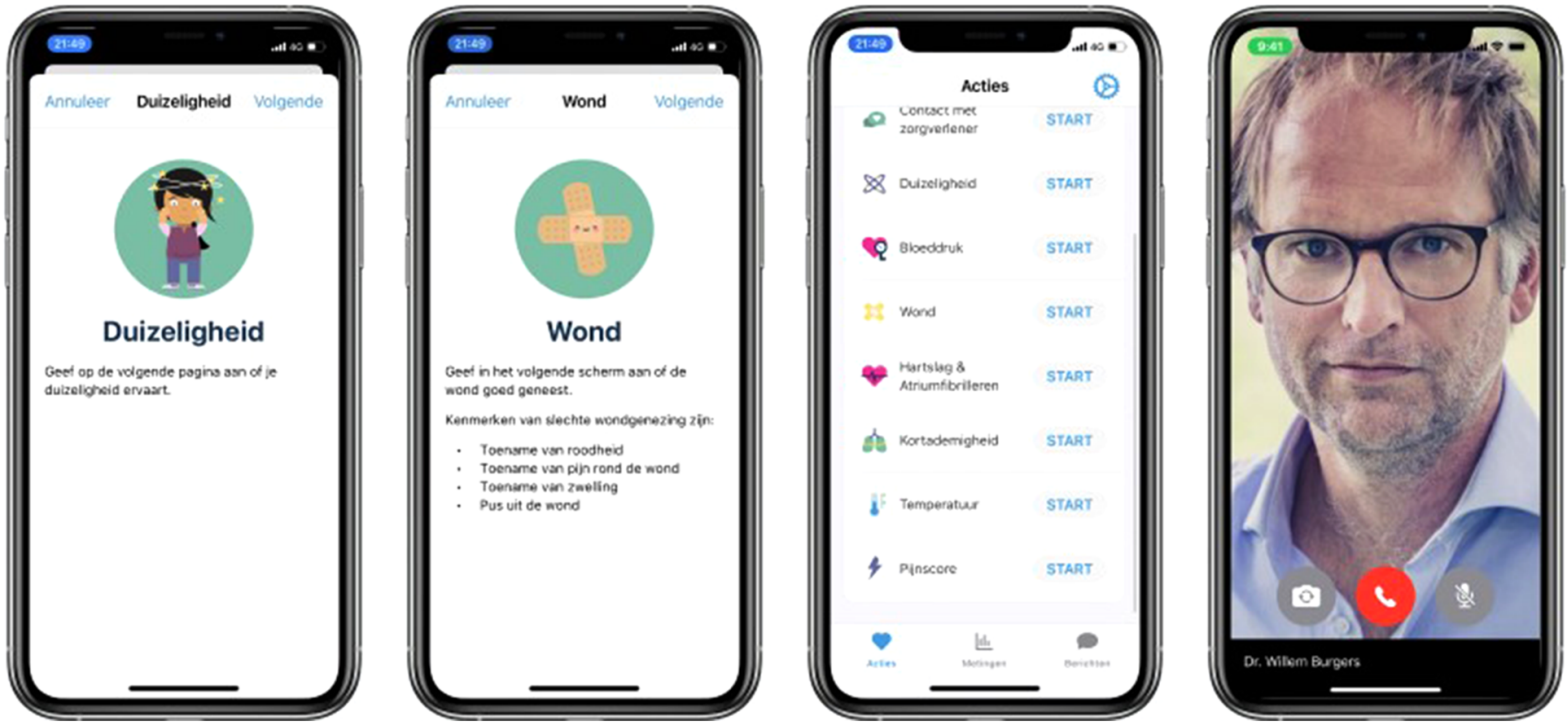
https://luscii.com/en/library/cardiothoracic-surgery.

The Amsterdam cardiac ecosystem harbors readiness to develop and clinically test innovation over the entire range of therapies. Patients who undergo cardiothoracic surgery need to be well prepared for surgery and the first week of follow-up after surgery is critical for a good recovery. For long-term follow-up the OLVG makes use of remote patient monitoring to guide patients after surgery with the help of a proprietary application that is hosted by a medical team e.g., patient care by means of wearables and interactive remote contact (Figure 9). The aim of the remote monitoring program is to help early patient discharge and reduce re-admissions. In addition, the program enables a faster titration of medication and reduces the number of unplanned calls after discharge. Patients are included on a pre-surgery protocol for baseline measurements and educational lessons to prepare the patient. In the first week after surgery, patients are monitored on a daily basis, using objective measurements such as weight, temperature, heart rate and blood pressure together with subjective questions over pain, dizziness and shortness of breath. Patients can also request contact with their medical team in case of uncertainty. After the first week, the protocol includes repeated measurements and questions on a weekly basis. After 4 weeks of monitoring, patients are referred to their own cardiologist, half of these cardiologists being from other hospitals.

It is questionable how convenient it may be to host all these innovation challenges under one roof, or even within a single organization. Besides the ambition of the professionals, the reimbursement system and the mission of the hospital, which extends beyond the cardiac center, significantly shape the conditions enabling innovation. In addition, referrals play an important role within this ecosystem, since much of postoperative care is conducted in outpatient clinics and secondary care facilities.

Costs, allocated budgets and over time cost-effectiveness of the novel mitral therapy are limiting factors. Competition for funding, resources and recognition by health care insurers within a hospital, region or country is a matter of fact and is driven by public interests and societal changes (80). For these reasons, readiness for innovation as a contribution to the evolution of cardiac care depends on a multifaceted organized and skilled cardiac center. However, the innovation should match the capability and societal profile of the center in order to yield individual and general patient benefits. In the early stages of marketing, the manufacturer used to pay for pre- and post-market approval studies, but the rising costs and the ethical issues related to the sponsoring of this kind of research have transformed the rules of the game. It is now evident from the complexity of mitral valve interventions that investigator-sponsored studies are indispensable as is shown by our literature references. The outcomes of such studies can act as a reality check in the sense that a new device with potentially promising outcomes may not be showing the expected results in real life (89), or they can confirm the complexity of a treatment such as TEER, resulting in its cautious application and valuable internal feedback for the institutional heart team (82). It is mandatory that medical specialists in the Amsterdam cardiac ecosystem continue to demonstrate a longstanding commitment to the patient beyond the friendly words of the clinical research assistants and nurse practitioners. The challenges of innovative treatments in sick patients with heart failure extend far beyond the brief period of admission and intervention. An infrastructure supported by wearables and Artificial Intelligence (AI) may enable low-threshold contact and bridge the “authority gap” between patient and doctor.

In summary, based on the experience in AUMC and OLVG, the boundary conditions for cardiac centers with the ambition to play a significant role in the evolution of the treatment of mitral valve disease include:

1. Transformation of innovation of technologies into processes and improved clinical outcomes.
2. Mutual commitment between researcher and patient to long-term follow-up.
3. Solid infrastructure for data collection and participation in studies that result in publications.
4. Awareness of a good quality of balanced risk communication with patients and environment, resulting in credible informed consent.

## Conclusion

The evolution of the surgical and transcatheter treatment of MR is not an isolated process but is embedded in various evolutionary tracks that are shaping cardiac care. The myriad and complexity of treatment processes, the need for institutional control and the interests of many stakeholders ensure a steady but slow evolution, despite worries about bureaucracy and professional competition. Innovations in devices and imaging cannot be detached from innovation in artificial intelligence to continuously monitor clinical data for improvement of IRMP. It all starts with operative skills, but improvement and attribution of skills in the areas of risk management and artificial intelligence are also mandatory in order to control the evolution of cardiac innovation.

The level and intensity of control are different: technology comes first in a transcatheter procedure, skills first in surgery.

The evolution of mitral valve interventions has taught us that current progress is based on applying complex technology which can only be safely used in a dedicated environment with sufficient attention to skills and risk control. Clinical practice either has been, or should be adjusted, to the adoption of new technology.

This process entails that the skills necessary to use the given technology should be addressed in the early development phase, in association with usability tests performed by clinical experts. In this phase, the training module and training platform can be created together with the assistance of proctors and product specialists. The learning curve related to all mitral interventions is steep and requires a sufficient amount of cases for heart teams and operators to develop and maintain skills and reduce risk.

An excellent IRMP is determined by the following conditions:

1. Excellent short- and long-term survival, low complications rate and a good quality of life for the treated patients.
2. Operating an effective system of data collection with appropriate interactions with referring cardiologists and patients during the treatment cycles with a low failure-to-rescue rate.
3. Transparency and publication of outcome date in the public domain and professional arena.
4. Maintaining adequate and cost-effective hospital infrastructure and resources enabling sufficient scale for skills development and number of interventions per operating team.

These conditions will redefine the boundaries of the traditional way of interpreting and practicing medicine and open the road to a new way of directing the therapeutic approach towards the treatment of mitral valve disease. According to today's analysis of the evolution, this requires a professional and institutional open-minded and “illuminated” vision on proven “old solutions” and new technological challenges.
